# Stromal Fibrosis of the Breast and the Associated Radiological Findings

**DOI:** 10.7759/cureus.15995

**Published:** 2021-06-28

**Authors:** Neel K Shroff, Flavia E Posleman Monetto

**Affiliations:** 1 Radiology, University of Texas Medical Branch, Galveston, USA

**Keywords:** stromal fibrosis, focal fibrosis, fibrous breast, mammogram, fibroadenoma

## Abstract

This case report follows a 42-year-old female patient who underwent a routine screening mammogram. The patient was found to have a 50 mm benign subareolar mass in the right breast. However, because benign imaging findings do not preclude malignancy, the patient was recommended to undergo a biopsy for confirmation. Subsequent imaging and core needle biopsy established a benign lesion consistent with stromal fibrosis with underlying fibroadenomatous changes. The benign imaging and histological findings of the breast mass were concordant. The patient was recommended yearly mammograms and continued observation. This case report highlights the importance of radiopathological concordance in patients found to have benign imaging findings on screening mammograms.

## Introduction

Stromal fibrosis is a benign breast lesion that may present as a clinically palpable mass or as an occult finding on routine screening mammography. Radiological characteristics of stromal fibrosis differ significantly making it a clinically difficult diagnosis. It can present with benign imaging findings such as an oval mass with circumscribed margins. These findings may not be followed up with biopsy resulting in missed diagnoses of underlying malignancies. In contrast, stromal fibrosis can exhibit malignant radiological features such as irregular, spiculated margins with calcifications. These conflicting characteristics are maintained across the imaging modalities of ultrasound, mammogram, and MRI. Due to their ambiguous nature, it is important to correlate radiologic evidence with histopathological findings to ensure no evidence of underlying malignancy.

## Case presentation

**Figure 1:**
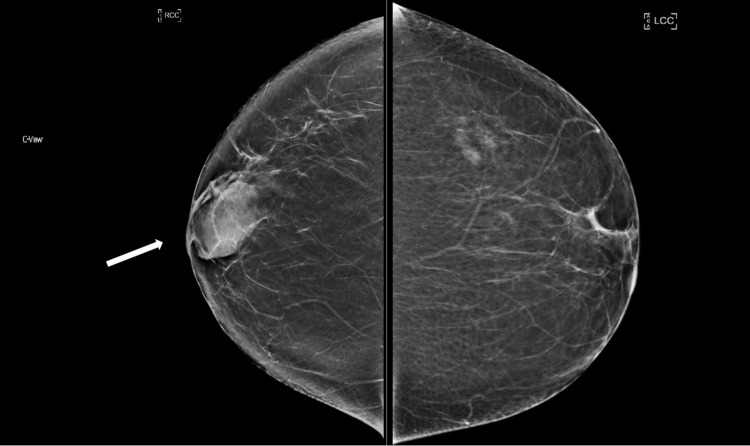
Right and left craniocaudal views. Bilateral craniocaudal views. There is a 50 mm isodense, oval mass with circumscribed margins in the right subareolar region (white arrow).

**Figure 2:**
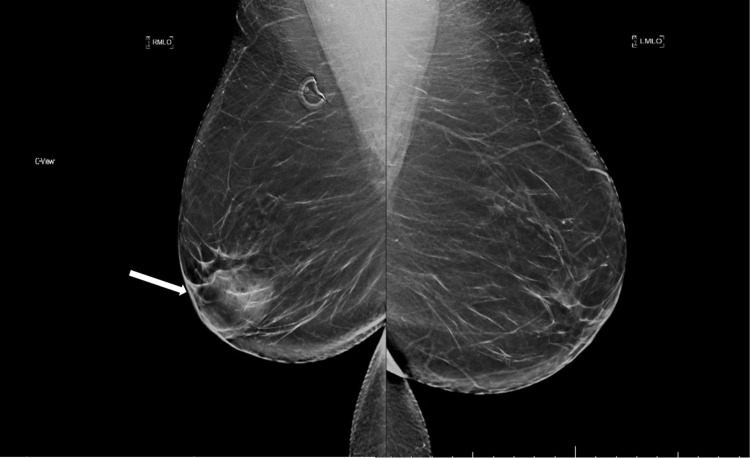
Right and left mediolateral oblique views. Bilateral mediolateral oblique views. There is a 50 mm isodense, oval mass with circumscribed margins in the right subareolar region (white arrow).

**Figure 3:**
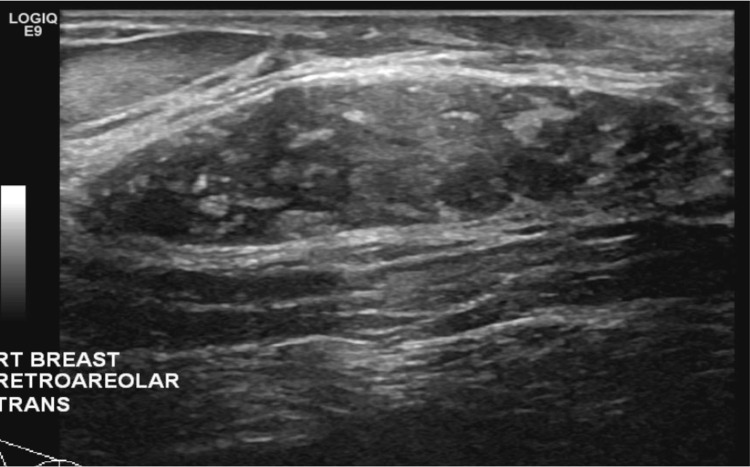
Ultrasound of the right breast (transverse view). Targeted ultrasound demonstrates an isoechoic, oval mass with circumscribed margins measuring 51 × 13 × 47 mm at the site of the mammographic mass in the subareolar region. Ultrasound of the axilla demonstrates morphologically normal lymph nodes.

**Figure 4:**
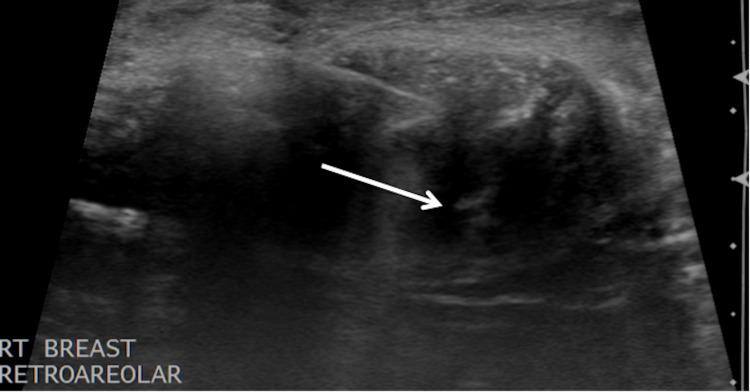
Core needle biopsy. Ultrasound-guided core needle biopsy of the right retroareolar mass. White arrowhead points at the biopsy needle.

**Figure 5:**
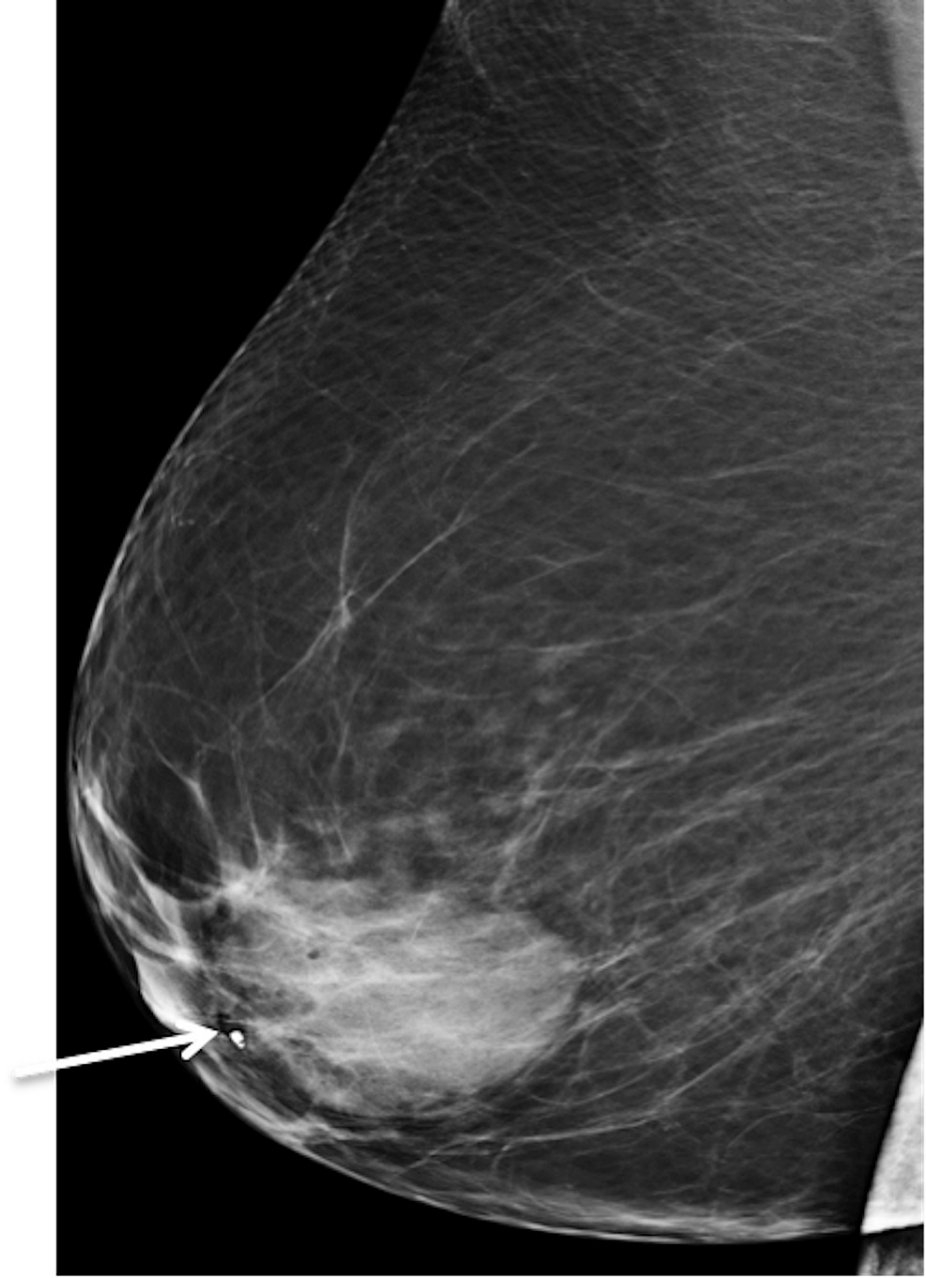
Post-biopsy image. Post-biopsy right lateromedial view with a coil marker clip (white arrow) at the edge of the retroareolar mass.

A 42-year-old female with a past medical history of Hashimoto’s thyroiditis underwent a routine screening mammogram. Prior to the mammogram, the patient had no complaints of a breast mass, breast tenderness, or breast discharge. The mammogram indicated a 50 mm circumscribed mass in the retroareolar region of the right breast with no evidence of calcifications or other malignant characteristics (Figures 1, 2). The finding was given a BI-RADS score of 0 and the patient was recommended to undergo ultrasound evaluation of the mass. Ultrasound of the right breast showed an isoechoic oval mass with circumscribed margins measuring 51 × 13 × 47 mm at the site of the mammographic mass in the subareolar region (Figure 3). Ultrasound of the axilla demonstrated morphologically normal lymph nodes. The finding was given a BI-RADS score of 4A and the patient was recommended to undergo ultrasound-guided core needle biopsy (Figures 4, 5). Subsequent biopsy of the mass demonstrated histopathological evidence of stromal fibrosis with fibroadenomatous changes. The image findings on mammogram and ultrasound exhibited benign characteristics. The patient’s histological findings consistent with stromal fibrosis and fibroadenomatous changes were concordant with the benign features on imaging. Thus, she was counseled on the benign nature of the mass and no requirement for surgical follow-up. The patient was scheduled for yearly mammograms to monitor for changes and was told to return if she noticed any changes or symptoms in the mass.

## Discussion

Fibrocystic change of the breast is a common condition that many women may develop during their lifetime [1]. These changes may occur in about 50% of all women [1]. Stromal fibrosis is a histopathological finding categorized as fibrocystic change [1]. It is a dense collagenous breast mass with little glandular or vascular tissue [2]. Although the disease typically occurs in premenopausal women, cases have been reported in postmenopausal women as well [2-5]. Stromal fibrosis is characterized by a histological finding of the proliferation of hypocellular fibrous stroma with destruction and atrophy of normal mammary ductal and acinar architecture [3,4,6-10]. Throughout the literature, stromal fibrosis has also been referred to as focal fibrosis, fibrous tumor, focal fibrous disease, or fibrous mastopathy [3,4,7,9,10].

Historically, stromal fibrosis has been defined as a clinically palpable mass commonly occurring in premenopausal women [2,3,4,5,7-11]. However, the emergence of screening mammograms has demonstrated that 65-100% of stromal fibrosis is clinically nonpalpable [3,4,7,8]. Several case series conducted via image-guided core needle biopsy have demonstrated a prevalence of 2-9% for stromal fibrosis, with one series indicating prevalence as high as 15% [3,4,6-12]. Radiologists must be able to identify and distinguish stromal fibrosis from malignant lesions due to its clinically occult nature, increasing prevalence, and nonspecific imaging characteristics.

Stromal fibrosis can be found as a clinically palpable mass or as an occult radiological finding. The etiology and pathogenesis of stromal fibrosis are not well understood. However, various mechanisms have been proposed for the development of the disease. One hypothesis is the proliferation of fibrous tissue as a result of hormonal stimulation from estrogen on fibroelastic tissue [3,4,7,8]. This theory is supported by the increased prevalence of stromal fibrosis in premenopausal women [4,7,8]. Furthermore, stromal fibrosis has also been reported in postmenopausal women on hormonal therapy, further supporting this theory [4]. Other hypotheses characterize the development of stromal fibrosis as the end stage of an inflammatory process or some type of breast involution [3,4,9].

Although it is a benign entity, stromal fibrosis is a leading cause of missed cases of breast cancer [3]. The presence of primary breast cancer is known to cause a desmoplastic proliferation of collagenous and fibrous tissue within the host [6]. Therefore, it is important to be cautious of a histopathological diagnosis of stromal fibrosis as there may be an underlying carcinoma adjacent to the tissue sampled [6]. The misdiagnosis of stromal fibrosis can be attributed to its wide spectrum of radiological findings ranging from benign to malignant characteristics [3,4,6-9]. Imaging modalities that can detect stromal fibrosis include ultrasound, mammograms, and breast MRI [3-6,8,11,12]. Stromal fibrosis has a myriad of radiological features specific to the imaging modality used and can vary from benign to malignant-appearing masses.

Many studies have been conducted to identify and document various lesion characteristics of stromal fibrosis due to overlapping benign and malignant features. For ultrasound imaging, studies have depicted varying characteristics for shapes, margins, echogenicity, and posterior enhancement. On ultrasound, stromal fibrosis can present with different shapes such as round, oval, irregular, or lobular [3,8-10]. The margins of stromal fibrosis are found to range from circumscribed to indistinct, angular, microlobulated, and spiculated [3,6-11]. Echogenicity also varies for stromal fibrosis, with most lesions presenting as hypoechoic masses and some lesions appearing hyperechoic, isoechoic, or complex [3,5,12]. Some cases of stromal fibrosis have been reported to demonstrate posterior acoustic shadowing or acoustic enhancement on sonographic imaging [3,5-10,12]. This constellation of findings makes it difficult to attribute a distinct pathognomonic imaging feature for stromal fibrosis on ultrasound.

Mammographic and sonographic imaging findings of stromal fibrosis are similar, ranging from benign to malignant characteristics. The most common finding for stromal fibrosis on mammography is an irregular mass or focal asymmetry [2,5]. Studies demonstrating mammographic findings of stromal fibrosis have shown varying shapes, margins, architectural distortion, and focal asymmetries [3,5,6,7,9,10]. Although rare, two studies have demonstrated findings of calcifications in areas of stromal fibrosis [3,5]. Some cases showed no evidence of stromal fibrosis on mammograms but displayed fibrosis after sonographic evaluation of the dense breast tissue [5,7]. Similar to ultrasound findings, mammographic evaluation of stromal fibrosis can display benign or malignant features requiring further diagnostic assessment.

In our patient, ultrasound imaging and mammography were sufficient in demonstrating stromal fibrosis. However, women with dense breast tissue may have mammographically and sonographically occult stromal fibrosis. Dense breast tissue can obscure findings on ultrasound and mammograms. In such cases, Lee et al. have demonstrated MRI as a useful tool for visualization of these lesions [4]. However, the pattern of collagen deposition in stromal fibrosis can obscure image characteristics on MRI as well [4]. Studies performed using MRI as a tool for stromal fibrosis have found a myriad of imaging characteristics. Lee et al. demonstrated most MRI findings of stromal fibrosis as small masses of round or oval shape with irregular or spiculated margins, rapid or medium rate of initial contrast uptake, and plateau or washout curves [4]. In contrast, Yilmaz et al. indicated an MRI finding of nonmass-like enhancement in most of their cases of stromal fibrosis. These lesions were found to have varying distributions: segmental, focal, or regional [8]. Some of these distribution patterns, such as segmental or linear, have a high positive predictive value for malignancy [4,8]. From the limited studies demonstrating imaging characteristics of stromal fibrosis on MRI, it is clear that there is not a distinct image finding for diagnosis. In many situations, stromal fibrosis can mimic carcinoma on MRI. Thus, there must be radiopathological concordance for an appropriate diagnosis of stromal fibrosis [3,4,6-12].

Due to the ambiguity in radiological findings for stromal fibrosis, pathological concordance is imperative to rule out underlying malignancy. Malik et al. demonstrated a 7% upgrade to malignancy on repeat biopsy of cases with histopathological evidence of stromal fibrosis [6]. Furthermore, Malik et al. described a 2% false-negative diagnosis of stromal fibrosis with image features concordant with a benign diagnosis. The diagnosis of stromal fibrosis must be confirmed by evidence from both image-guided biopsy as well as radiologic findings. Benign radiological findings on imaging along with a histological finding consistent with stromal fibrosis can be managed conservatively because the two findings corroborate each other. However, malignant characteristics on radiological imaging with histological evidence of stromal fibrosis need further testing because the result is equivocal and malignancy cannot be ruled out. These cases of radiopathological discordance are recommended to undergo subsequent repeat biopsy or surgical excision to ensure removal of possible malignancy [3,4,6-11]. Although our patient did not demonstrate malignant findings on screening mammogram, it was important to ensure that there was no underlying malignancy as there have been cases demonstrating malignant pathological findings in patients with benign imaging characteristics.

## Conclusions

Stromal fibrosis is a benign condition of the breast routinely found on screening mammograms with no clinically palpable mass. Radiologically, the lesion has variable findings across modalities such as ultrasound, mammogram, and MRI. Image presentation of the disease can vary in shape, margin, calcification, and echogenicity. These inconsistencies make it difficult to distinguish this benign condition from malignancy. Histologically, the disease shows benign findings of proliferative fibrous stroma and destruction of normal mammary architecture.

Concordance between radiological and histological findings is essential to rule out malignancy. In cases of radiopathological discordance, further biopsy or surgical excision should be performed to ensure the removal and treatment of potentially malignant lesions. In our patient, benign imaging features supported the benign histopathological findings of stromal fibrosis, and surgical intervention was not warranted. It is important that further data regarding imaging characteristics of stromal fibrosis are collected to improve radiology training in distinguishing stromal fibrosis from potentially malignant lesions.
